# Supplementary material for: Causal Relationship between Aging and Anorexia Nervosa: A White-Matter-Microstructure-Mediated Mendelian Randomization Analysis
Source: Biomedicines. 2024 Aug 16;12(8):1874. doi: 10.3390/biomedicines12081874 (PMC11351342; doi:10.3390/biomedicines12081874)
Supplement: Supplementary file 1 [file biomedicines-12-01874-s001.zip › Supplementary Figure/Figure S4/Singe SNP plot of MR analysis for TL to ubm-b-1576.pdf]

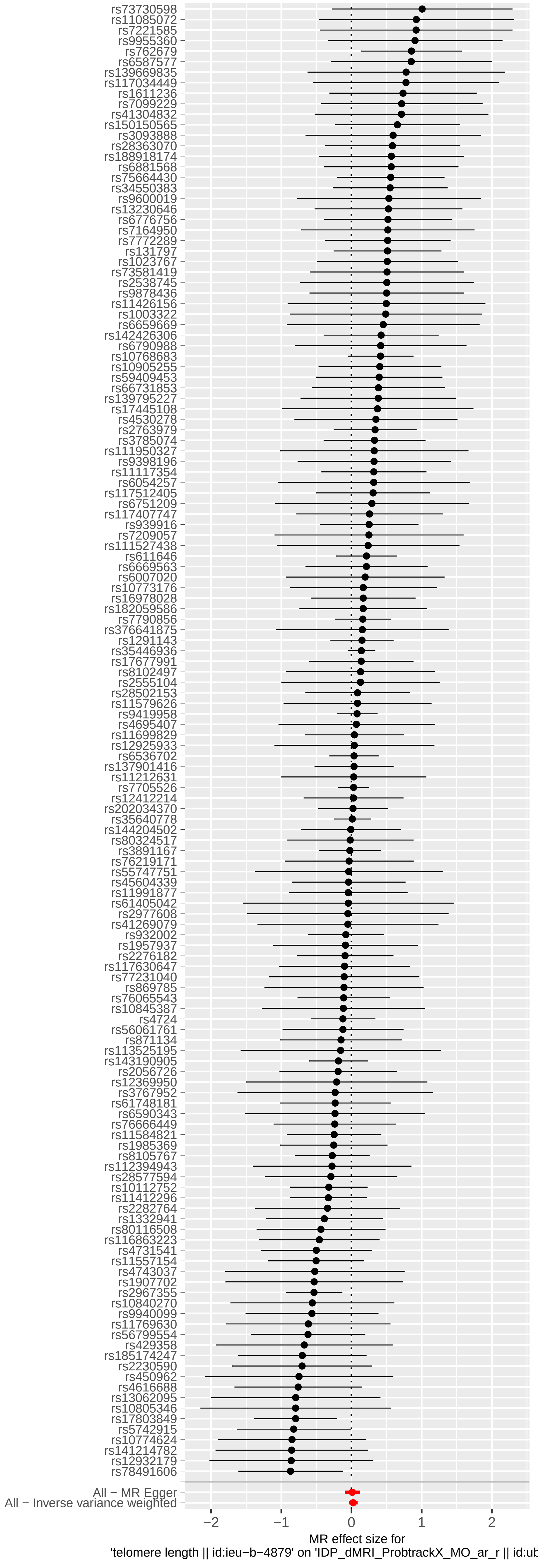
rs73730598
rs11085072
rs7221585
rs9955360
rs762679
rs6587577
rs139669835
rs117034449
rs1611236
rs7099229
rs41304832
rs150150565
rs3093888
rs28363070
rs188918174
rs6881568
rs75664430
rs34550383
rs9600019
rs13230646
rs6776756
rs7164950
rs7772289
rs131797
rs1023767
rs73581419
rs2538745
rs9878436
rs11426156
rs1003322
rs6659669
rs142426306
rs6790988
rs10768683
rs10905255
rs59409453
rs66731853
rs139795227
rs17445108
rs4530278
rs2763979
rs3785074
rs111950327
rs9398196
rs11117354
rs6054257
rs117512405
rs6751209
rs117407747
rs939916
rs7209057
rs111527438
rs611646
rs6669563
rs6007020
rs10773176
rs16978028
rs182059586
rs7790856
rs376641875
rs1291143
rs35446936
rs17677991
rs8102497
rs2555104
rs28502153
rs11579626
rs9419958
rs4695407
rs11699829
rs12925933
rs6536702
rs137901416
rs11212631
rs7705526
rs12412214
rs202034370
rs35640778
rs144204502
rs80324517
rs3891167
rs76219171
rs55747751
rs45604339
rs11991877
rs61405042
rs2977608
rs41269079
rs932002
rs1957937
rs2276182
rs117630647
rs77231040
rs869785
rs76065543
rs10845387
rs4724
rs56061761
rs871134
rs113525195
rs143190905
rs2056726
rs12369950
rs3767952
rs61748181
rs6590343
rs76666449
rs11584821
rs1985369
rs8105767
rs112394943
rs28577594
rs10112752
rs11412296
rs2282764
rs1332941
rs80116508
rs116863223
rs4731541
rs11557154
rs4743037
rs1907702
rs2967355
rs10840270
rs9940099
rs11769630
rs56799554
rs429358
rs185174247
rs2230590
rs450962
rs4616688
rs13062095
rs10805346
rs17803849
rs5742915
rs10774624
rs141214782
rs12932179
rs78491606
All − MR Egger
All − Inverse variance weighted
−2
−1
0
1
2
MR effect size for
'telomere length || id:ieu-b-4879' on 'IDP_dMRI_ProbtrackX_MO_ar_r || id:ub
